# Broader Geographical Distribution of Toscana Virus in the Mediterranean Region Suggests the Existence of Larger Varieties of Sand Fly Vectors

**DOI:** 10.3390/microorganisms8010114

**Published:** 2020-01-14

**Authors:** Nazli Ayhan, Jorian Prudhomme, Lison Laroche, Anne-Laure Bañuls, Remi N. Charrel

**Affiliations:** 1 Unité des Virus Emergents (Aix-Marseille Univ–IRD 190–Inserm 1207–IHU Méditerranée Infection), 13005 Marseille, France; remi.charrel@univ-amu.fr; 2 Unité de Virologie EA7310 Bioscope, Université de Corse Pasquale Paoli (UCPP), 20250 Corte, France; 3 UMR MIVEGEC (IRD—CNRS—Université de Montpellier), 911 avenue Agropolis, F34394 Montpellier, France; lisonlaroche@hotmail.fr (L.L.); anne-laure.banuls@ird.fr (A.-L.B.)

**Keywords:** Toscana virus, sand fly, *Phlebotomus*, *Sergentomyia*, Mediterranean area, *Phenuiviridae*, Bunyavirales, *Sandfly fever Naples phlebovirus*, arbovirus, arthropod-borne, sandfly, phlebotomine

## Abstract

Toscana virus (TOSV) is endemic in the Mediterranean basin, where it is transmitted by sand flies. TOSV can infect humans and cause febrile illness as well as neuroinvasive infections affecting the central and peripheral nervous systems. Although TOSV is a significant human pathogen, it remains neglected and there are consequently many gaps of knowledge. Recent seroepidemiology studies and case reports showed that TOSV’s geographic distribution is much wider than was assumed a decade ago. The apparent extension of the TOSV circulation area raises the question of the sandfly species that are able to transmit the virus in natural conditions. *Phlebotomus (Ph.)* *perniciosus* and *Ph. perfiliewi* were historically identified as competent species. Recent results suggest that other species of sand flies could be competent for TOSV maintenance and transmission. Here we organize current knowledge in entomology, epidemiology, and virology supporting the possible existence of additional phlebotomine species such as *Ph. longicuspis, Ph. sergenti, Ph. tobbi, Ph. neglectus, and Sergentomyia minuta* in TOSV maintenance. We also highlight some of the knowledge gaps to be addressed in future studies.

## 1. Introduction

The genus *Phlebovirus* includes 58 viruses classified into a ten-species complex: Bujaru, Candiru, Chilibre, Frijoles, Punta Toro, Rift Valley fever, Salehabad, Sandfly fever Naples, Severe fever with thrombocytopenia syndrome, and Uukuniemi phleboviruses. The Sandfly fever Naples phlebovirus species comprises thirteen viruses, including Toscana virus (TOSV) [1]. TOSV has a tropism for central and peripheral nervous systems and is responsible for meningitis and encephalitis in the Mediterranean region [2].

Phleboviruses are transmitted to humans by the bite of an infected female sand fly during the blood meal. There is still little information on the development cycle of phleboviruses in the vector [3].

During the last decade, the known geographical area where TOSV circulates has increased considerably. With recent entomological studies, seroepidemiological studies, and case reports, the known distribution of TOSV is now extended in Central and Eastern Europe, North Africa, and Turkey.

We suggest that the existence of a larger diversity of sand fly vectors may explain the TOSV circulation in different geographical regions, which is supported by the recent identification of the virus in phlebotomine species that were not previously considered as TOSV vectors.

Here we present a comprehensive analysis of recent data suggesting that additional species of sand flies are involved in natural cycles to explain the recently revealed broader geographical distribution of TOSV in the Mediterranean region.

## 2. Toscana Virus

### 2.1. Overview on Toscana Virus

TOSV is an enveloped, tri-segmented RNA arbovirus which belongs to the genus *Phlebovirus*, the family *Phenuiviridae*, and the order Bunyavirales [4]. At the family level, genomes are comprised of three unique molecules of negative or ambisense single-stranded RNA, designated L (large), M (medium), and S (small) to a total of 11 to 19 kb. Within each genus, viruses share similar segment and structural protein sizes, as well as characteristic terminal sequences at the 3’ and 5’ ends of each of the three segments. TOSV distribution and abundance are highly dependent on its arthropod vector. Human cases are observed during the warm season, with a peak during the hottest months in relation with sand fly activity. This virus shows a peculiar neurotropism and causes central nervous system (CNS) diseases in infected individuals [2,5]. Like other arboviruses, most TOSV infections in humans are asymptomatic [6]. So far, 864 symptomatic human cases (834 autochthonous and 30 imported) have been reported in residents of different Mediterranean countries and in tourists and travelers returning from endemic regions [7,8,9,10].

**Figure 1:**
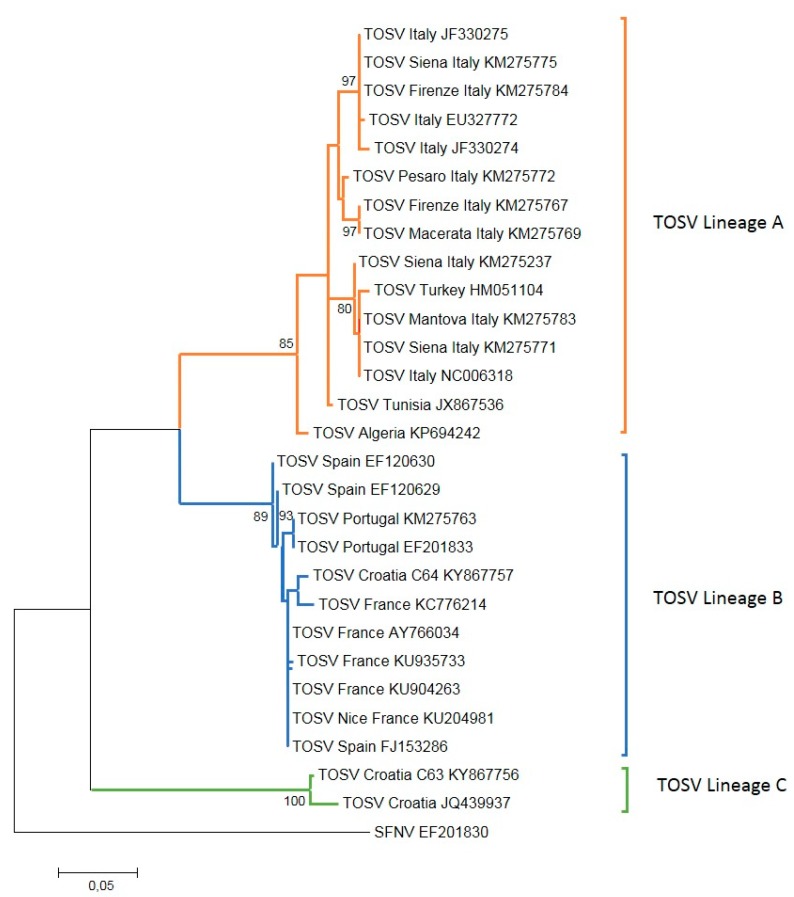
Phylogenetic analysis of Toscana virus (TOSV) based on the nucleocapsid protein gene. Maximum likelihood analysis at nucleotide level was performed using the MEGA 6.06 software program [15]. Bootstrap values are indicated and correspond to 1000 pseudoreplications.

Genetic studies have identified the existence of three lineages of TOSV (A, B, and C) [11] (Figure 1). To date, there is no difference in virulence or clinical manifestations depending on the genetic lineage. At least two lineages co-circulate in France [12], Turkey [13], and Croatia [14]. Further studies are required in order to have more information about the genotype distribution and their association with vector distribution. The known geographical distribution of TOSV in the Mediterranean basin raises the question of whether the demonstrated vectors, *Phlebotomus (Ph.) perniciosus* and *Ph. perfiliewi*, can explain this distribution or whether other species could be implicated.

The knowledge of the vectors and geographical distribution of TOSV is increasing in parallel with the number of studies. The current TOSV distribution includes South European countries (Italy, Spain, France, and Portugal) [16,17,18,19], East Mediterranean countries (Turkey and Cyprus) [13,20], Balkan countries (Greece, Croatia, Bosnia and Herzegovina, Kosovo, and Bulgaria) [21], and North African countries (Tunisia, Morocco, and Algeria) [22]. The geographical distribution of TOSV in the Mediterranean basin raises the following question: Can the known vectors, *Ph. perniciosus* and *Ph. perfiliewi*, explain this distribution, or are other species implicated?

### 2.2. Epidemiology of Toscana Virus

**Figure 2:**
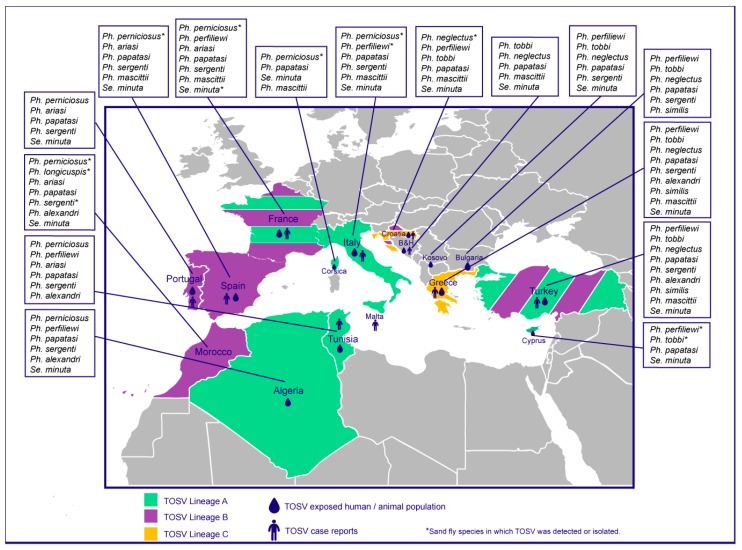
Geographical distribution of Toscana virus (TOSV) and reported distribution of predominant sand fly species in the countries where TOSV is present.

Seroprevalence studies demonstrate that human populations are exposed to TOSV in Italy, Spain, France, Portugal, Turkey, Malta, Cyprus, Greece, Croatia, Bosnia and Herzegovina, Kosovo, Bulgaria, Tunisia, and Algeria [7,22,23,24,25,26,27,28,29,30,31,32,33,34,35,36]. Together, the results of serological surveillance studies and case reports showed that the TOSV geographic distribution is much wider than believed a decade ago; in particular, the presence of TOSV has been recently documented in North Africa and the Balkan countries [31,32,34,35,36] (Figure 2).

High rates of TOSV-neutralizing antibodies (17.2–59.4%) have been identified in the healthy population of Tunisia, with geographical variation [35,36]. Similar results were obtained in Algeria with a prevalence up to 50% in Draa El Mizan [22]. Although the studies are geographically limited within Tunisia and Algeria, the high rates of neutralizing antibodies emphasize the massive circulation of TOSV in these countries (Figure 2).

In Kosovo, 5.5% of blood donors possess IgG against TOSV [32]. Higher rates were reported in healthy residents of Croatia, with the highest values in coastal areas and Croatian islands [31]. Another study identified the presence of TOSV antibodies in patients from Bosnia and Herzegovina [33]. Recently, anti-TOSV IgGs were found in 24.4% of healthy residents of Bulgaria [34] (Figure 2).

### 2.3. Pathways of TOSV Transmission and Maintenance

Transovarial and venereal pathways of TOSV transmission have been demonstrated experimentally with *Ph. perniciosus* colonies [37,38,39]. These experimental results were complemented by the observation in nature of equal rates of TOSV infection in male and female sand flies, suggesting the existence of transovarial (vertical) and/or venereal (horizontal) transmission during mating [40,41,42,43]. Additionally, the survival of TOSV in overwintering *Ph. perniciosus* larvae underlined the capability of TOSV for maintenance during diapause [37]. Co-feeding transmission of TOSV during sugar meal was also suggested experimentally in males and females since it was demonstrated in *Ph. perniciosus* with Massilia virus, a relative of TOSV also transmitted by *Ph. perniciosus* in nature [44]. Future studies need to be conducted with TOSV and other species belonging to the *Phlebotomus* genus in order to confirm that viral transmission is possible with other species.

## 3. Sand Flies

### 3.1. Overview on Sand Flies

Sand flies are tiny blood-feeding (hematophagous) insects belonging to the order Diptera, family Psychodidae, and subfamily Phlebotominae. Approximately 900 sand fly species are described, of which 70 have been identified as potential vectors of *Leishmania* [45] and a few species were associated with *Phlebovirus* and other viruses [46]. Each species can be identified by the characteristic shape of their cibarium, pharynx, and reproductive organs [47].

Both genders feed on honeydew, plant sap, and aphid secretions [48], but only females take a blood meal, necessary for egg maturation. Sand flies present a crepuscular and nocturnal activity [49]. They are very abundant in warm regions (e.g., Mediterranean basin, Asia, Africa, South America), but their range is very wide (between 50° N and 40° S). They occur on all continents but have not been reported in New Zealand or the Pacific Islands. Their altitudinal distribution ranges from sea level to 3500 m above sea level (Afghanistan: *Ph. rupester*) [50,51,52]. Modeling studies suggest an expansion of the range of different *Phlebotomus* species in Europe due to the influence of environmental and climate change (*Ph. ariasi*, *Ph. mascittii*, *Ph. perniciosus*, *Ph. neglectus,* and *Ph. perfiliewi*) allowing the colonization of new habitats [53,54].

However, this insect remains relatively unknown from a biological and ecological point of view (unknown breeding sites, poorly known food preferences, etc.).

### 3.2. Sandflies as TOSV Vectors

Sandfly activity is seasonal in the Mediterranean countries. They are present from May to October due to low temperatures in winter. The average annual temperatures and the latitude influence the emergence periods and the seasonal dynamics of these insects (with a peak in July and August) [49]. Their activity and abundance are dependent on environmental and climatic conditions and may vary depending on the species and the location of capture.

The first isolation of TOSV was obtained from *Ph. perniciosus* in central Italy, in 1971 [55]. Subsequently, other strains were isolated from *Ph. perfiliewi* [56]. The isolation or detection of TOSV in sand flies started to be reported not only in Italy but also in other Mediterranean countries. *Ph. perniciosus* was identified as vector species of TOSV in Southern France [12], the South-West region of Madrid, Spain [57], and Morocco [58]. Additionally to *Ph. perniciosus, Ph. longicuspis* and *Ph. sergenti* are identified as potential vectors of TOSV in Morocco [59]. Another study reported TOSV presence in *Se. minuta* in France which feeds preferentially on cold-blooded vertebrates [60]. More recently, TOSV was detected from two pools of *Ph. neglectus* in Croatia [14]. In Cyprus, TOSV was detected in one pool of *Ph. perfiliewi* and in tow pools of *Ph. tobbi* [61].

In some other studies, the infected vectors were identified at the genus level only, thus preventing implication of a given species. This was the case in Spain (where almost 70% of captured insects were *Ph. perniciosus)* [62], Corsica (where *Ph. perniciosus* and *Se. minuta* were largely predominant in equal proportions) [63], Algeria (where six species were morphologically identified: *Ph. perfiliewi* (51.4%), *Ph. perniciosus* (36.7%), *Ph. longicuspis* (2.6%), *Ph. papatasi* (6.5%), *Ph. sergenti* (0.5%), and *Se. minuta* (2.3%)) [22], and in Tunisia (where *Ph. perniciosus* was the most abundant species (71.74%)) [64] (Figure 2).

To date, only TOSV lineage A has been recorded in *Ph. perfiliewi* [56]. However, at this stage of knowledge, the correlation of TOSV genetic lineage and phlebotomine species is poorly supported (Figure 2). *Ph. perniciosus* can transmit TOSV strains belonging to lineage A and lineage B [12,55,57,58]. Lineage A was also detected once in *Se. minuta* [60]. In addition to *Ph. perniciosus, Ph. longicuspis, Ph. sergenti,* and *Ph. neglectus* are possible vectors of TOSV strains belonging to lineage B [11,59]. So far, TOSV strains belonging to lineage C have only been recorded in Croatia and Greece, where they are assumed to be transmitted by *Ph. neglectus* [14] (Figure 2).

In regions where known vector species (e.g., *Ph. perniciosus* and *Ph. perfiliewi*) are in the minority or even totally absent (e.g., Bosnia and Herzegovina), seroprevalence rates observed in humans and animals suggest that alternative species could be competent (e.g., *Ph. neglectus* in Bosnia and Herzegovina). It is therefore important to study the specificities for each country and species in order to better understand the current expansion of TOSV together with its demonstrated and putative vectors.

Climate change affects the geographical distribution of many sand fly species [65,66]. As a consequence, the geographical distribution of sand flies has expanded towards Northern Europe during the last decade. For instance, populations of *Ph. perniciosus* are now present in new regions in Germany [54,67]. Consequently, TOSV could also spread to these newly colonized regions where competent species are present [68].

Despite the lack of knowledge for many species of sand flies, those belonging to the genus *Phlebotomus* have benefited from recent efforts from the European Center for Disease Prevention and Control (ECDC) in terms of species inventory and mapping. However, these efforts should be pursued and completed by abundance studies [69]. Regarding *Sergentomyia* species, very little is known, nevertheless these species deserve more attention due to their possible association with TOSV transmission [60] and their putative role in *Leishmania* transmission [70,71,72,73].

## 4. Vertebrates as Possible Reservoir or Amplification Host

The conditions required for a given species to be an efficient reservoir or amplifying host are (i) to be able to generate high and/or sustained viremia for transmission to the competent sand fly species and (ii) to present a geographical distribution equal to, or larger than, that of the disease.

In humans the short duration of viremia together with the absence of persistent infection preclude any role in TOSV maintenance in nature. TOSV was isolated once from the brain of one wild caught bat (*Pipistrellus kuhlii*) in Italy where TOSV-infected *Ph. perniciosus* and *Ph. perfiliewi* were present [56]. This is the first and the only record of TOSV identification from bats.

Many seroprevalence studies testing vertebrate sera have described the presence of variable rates of antibodies directed against TOSV. These studies showed that TOSV circulates in the following countries: Greece [74], Spain [75], Portugal [76,77], France (Corsica island) [78], Tunisia [79], Algeria [80], and Kosovo [81], and can infect dogs, cats, cattle, and sheep. One study reported the presence of TOSV RNA detection in dogs in Portugal [76] and another study detected TOSV RNA in goat serum in southern Spain [75]. These results are very likely to be anecdotic. So far, there is no evidence for a vertebrate species acting as reservoir or amplifying host in the natural cycle of TOSV. Considering that dogs are a reservoir for sand-fly-borne *Leishmania infantum*, they are good candidates as natural reservoir hosts for TOSV. The fact that neutralizing antibodies against TOSV have been repeatedly described in dogs (4.3–8.4% in studies conducted in France, Portugal, Tunisia, Algeria, Greece, and Cyprus) is likely to reflect exposure rather than being indicative of a more peculiar role in the natural cycle [74,76,77,78,79,80]. However, two studies conducted in dogs from Mediterranean Anatolia (Turkey) reported the presence of TOSV RNA in the blood of approximately 3% and 10% of tested dogs, respectively, of which some were co-infected with *Leishmania* parasites [82,83]. Co-infection could contribute to this apparent active replication of TOSV. More recently, TOSV was detected in the brain and kidney from a greater flamingo (*Phoenicopterus roseus*), a great white pelican (*Pelecanus onocrotalus*), and a black stork (*Ciconia nigra*) in Turkey [84]. Nevertheless, the role of these animals in the life cycle of TOSV remains unknown. Currently, no data support the hypothesis that humans and/or any other vertebrates are the reservoirs of sand-fly-borne phleboviruses, due to the short duration of the viremia and the lack of persistent infection. The consideration of the vector as the reservoir of phleboviruses is currently under debate [60].

## 5. Conclusions

Here we highlight that there is very limited information on the biology and epidemiology of TOSV, its reservoirs, and its vectors. As there is neither a vaccine nor a specific treatment, the control of TOSV infections can only be achieved through sand fly control measures (indoor and outdoor residual spraying, attractive toxic sugar baits, etc.) or personal protection against bites (skin repellents, impregnated bed nets, etc.). In order to be able to target regions where these measures could be promoted, it is necessary to know the geographical distribution of populations at risk through seroepidemiological studies and the surveillance of neuro-invasive TOSV infection cases. The recent demonstration that TOSV circulates in a much broader area than suspected a decade ago has raised questions about the possibility that additional phlebotomine species (at least *Ph. longicuspis, Ph. sergenti, Ph. tobbi, Ph. neglectus, and Sergentomyia minuta)* may be involved in the TOSV natural cycle. Whether these species or others could be competent for the transmission of TOSV merit further study. Accordingly, it is necessary to set up specific studies to address this question (i) in the natural environment and (ii) under experimental conditions using sand fly colonies in insectarium.
